# Supplementary material for: Predictive Utility of Biochemical Markers for the Diagnosis and Prognosis of Gestational Diabetes Mellitus
Source: Int J Mol Sci. 2024 Oct 30;25(21):11666. doi: 10.3390/ijms252111666 (PMC11545977; doi:10.3390/ijms252111666)
Supplement: Supplementary file 1 [file ijms-25-11666-s001.zip › ijms-3272905-supplementary.pdf]

International Journal of
Molecular Sciences

MDPI

Supplementary data

Table S1. Biochemical markers for the diagnosis of gestational diabetes mellitus (GDM).

Biomarker	Trimester	Cut-off	Effect in GDM	Sens.	Spec.	AUC	Reference
Adipokines							
Adiponectin [µg/mL]	1st	-	↓	-	-	0.634	[51] [52] [53]
	2nd	8.482	↓	81.11	82.14	0.801	[54] [55]
Leptin [ng/mL]	1st	18.9	↑	95.7	68.6	0.601 - 0.812	[51] [56]
	2nd	8.5 - 11.43	↑	83.3 - 90	72.1 - 96	0.956	[57] [58]
Chemerin [ng/mL]	1st	-	↑	-	-	0.581	[51]
	2nd	6.78 - 15.49	↑	73.33 - 96	72 - 76	0.820 - 0.970	[57] [59]
FABP4 [ng/mL]	1st	18.5	↑	81.8	71.2	0.733	[60]
	2nd	1.96 - 27.64	↑	75.00 - 86.96	80.00 - 89.09	0.814 - 0.940	[59] [61]
Irisin [ng/mL]	1st	149 - 540	↓	66.7 - 90	70.7 - 100	0.723 - 0.940	[62] [63]
	2nd	484.74	↓	85	62.5	0.753	[64]
Retinol binding protein 4 (RBP4) [µg/mL]	1st	23.4	↑	85.2	53.1	0.737	[65]
	2nd	34.84	↑	79.4	79.1	0.87	[66]
Betatrophin (ANGPTL8) [pg/mL]	1st	2792.33	↑	54.55	79.27	0.706	[67]
	2nd	0.95 x 106	↑	68.8	84.1	0.812	[68]
Resistin (ng/mL)	1st	5.3	↑	95.7	61.4	0.836	[56]
Secreted frizzled-related protein 5 (SFRP5) [ng/mL]	1st	12.72	↓	62.5	88.64	0.824	[69]
Visfatin (ng/mL)	1st	2.8	↑	87.1	70	0.799	[56]
C1q/TNF-Related Protein 9 (CTRP9) [pg/mL]	1st	-	↓	-	-	0.776	[70]
Fetuin-A (pg/mL)	1st	305.9	↑	64.4	58.5	0.612	[71]
Secreted frizzled-related protein 4 (SFRP4) [ng/mL]	1st	-	↑	-	-	0.605	[51]
Asprosin [ng/mL]	2nd	31.709	↑	93.3	90.9	0.970	[72]
FGF-21 [ng/mL]	2nd	82.07	↑	100	85	0.95	[73]
Lipocalin 2 (LCN2) [ng/mL]	2nd	23.69	↑	86.67	71.62	0.887	[54]
Omentin-1 [ng/mL]	2nd	27.33 - 244.14	↓	81 - 99.30	78 - 96.70	0.836 - 0.854	[74] [75]
C1q/TNF-Related Protein 3 (CTRP3) [ng/mL]	2nd	-	↓	-	-	0.837	[76]

**Biomarker**	**Tri-mester**	**Cut-off**	**Effect in GDM**	**Sens.**	**Spec.**	**AUC**	**Reference**
Myonectin or C1q/TNF-related protein 15 (CTRP15) [ng/mL]	2nd	57.62	↓	82.5	72.5	0.824	[64]
Adropin [ng/mL]	2nd	3.75	↓	67.7	78.8	0.722	[77]
Pentraxin 3 (PTX3) [ng/mL]	2nd	-	↑	94.9	32.8	0.72	[78]
Neuregulin 4 (NRG4) [ng/mL]	2nd	96.25	↓	66.67	62.07	0.626	[79]
Inflammatory markers							
IL-6 [pg/mL]	1st	-	↑	51.3	-	0.673	[52] [53] [80]
	2nd	-	↑	-	-	-	[52] [53]
TNF-alpha [pg/mL]	1st	-	↑	-	-	-	[53] [81]
	2nd		↑	-	-	-	[53] [82]
hs-CRP [mg/L]	1st	4.65	↑	86.21	50.85	0.702	[83]
	2nd	2.28 - 3.09	↑	77.30 - 85	72 - 94.70	0.856 - 0.89	[74] [75]
Fibrinogen [g/L]	2nd	2.80	↑	86.70	85.14	0.87	[74]
Insulin resistance markers							
HOMA-IR	1st	1.80 - 2.08	↑	79.1 - 90	56.3 - 61	0.703 - 0.809	[84] [85]
	2nd	2	↑	94.5	72.2	0.913	[86]
SHBG [nmol/L]	1st	158	↓	81.5	80.1	0.874	[87]
	2nd	452	↓	80.1	84.2	0.897	[88]
TyG index	1st	7.088 - 8.890	↑	61.7 - 68.2	61.7 - 64.3	0.641 - 0.692	[84] [89]
TyHGB	1st	6.16	↑	57	70.3	0.682	[90]
QUICKI	2nd	0.34	↓	86.4	83.3	0.905	[86]
C-peptide	2nd	-	↑	-	-	0.764	[91]
Insulin	2nd	-	↑	-	-	0.714	[91]
Glycemic markers							
HbA1c [%]	1st	5.33 – 5.45	↑	54.8 - 83.3	69.0 - 96.8	0.809 – 0.840	[92] [93] [94]
	2nd	5.45 -5.7	↑	73.3 – 84.3	75.6 – 81.8	0.826 – 0.848	[95] [96]
FPG [mg/dL]	1st	81 – 88.5	↑	64.29 – 79.31	56.45 – 59.32	0.63 – 0.738	[83] [97] [98]
	2nd	81.9	↑	-	-	0.712	[99]
1,5-AG [μg/mL]	2nd	13.21	↓	67.6	65.3	0.693 – 0.722	[100]
pGCD59 [SPU]	2nd		↑	-	-	0.65	[101] [102]
Glycated albumin (GA) [%]	2nd	-	↑	-	-	0.568	[103]

Biomarker	Trimester	Cut-off	Effect in GDM	Sens.	Spec.	AUC	Reference
Fructosamine [μmol/L]	2nd	222	↑	54.8	48.6	0.52	[104]
Lipid profile markers							
TG [mmol/L]	1st	1.235 - 2.375	↑	73.7 - 86.27	59.3 - 66.67	0.622 - 0.813	[105] [106] [107]
	2nd	1.525 – 2.66	↑	72.09	71.6	0.587 - 0.769	[106] [108]
TG/HDL-C	1st	0.831 - 2.2684	↑	63.7 - 72.97	64.3 - 75.05	0.664 - 0.786	[84] [109]
	2nd	1.12 - 4.254	↑	73.7 - 79.07	56.9 - 78	0.705	[108] [110]
Remnant cholesterol [mg/dL]	1st	24.30	↑	86.49	64.20	0.8038	[111]
Atherogenic index of plasma	1st	0.3557	↑	72.22	75.41	0.7879	[112]
LDL-C/HDL-C	1st	0.928	↑	66.7	46.2	0.574	[84]
Placenta-derived factors							
PlGF [MoM]	1st	0.89	↑	51.2	87.2	0.68	[113]
PAPP-A [MoM]	1st	0.885	↓	66.67	65.50	0.654	[114]
βhCG [MoM]	1st	0.990	↓	74.40	46.80	0.603	[114]
Myostatin [ng/mL]	2nd	-	↑	92.5	65.6	0.84	[78]
sFlt-1 [ng/mL]	2nd	-	↑	94.9	42.6	0.70	[78]
Follistatin (FST) [ng/mL]	2nd	-	↑	92.3	57.4	0.67	[78]
Placental Protein 13 [pg/mL]	2nd	-	↑	92.3	62.3	0.63	[78]
Metabolic markers							
s(Pro)RR [ng/mL]	1st	24.52	↑	75	80	0.82	[115]
	2nd	23.3	↑	68	70	0.828	[116]
Zonulin [ng/mL]	1st	6.27	↑	76.8	75	0.79	[117]
	2nd	12.71 – 20	↑	72.7 - 78.95	63.64 - 71.4	0.755 - 0.796	[117] [118]
25-(OH)D [ng/mL]	1st	6.0 - 23.14	↓	64.71 - 81.0	44.0 - 70.59	0.67 - 0.721	[107] [119]
	2nd	14.0	↓	-	-	0.66	[120]
Osteocalcin [ng/mL]	1st	-	↑	-	-	0.61	[121]
Homocysteine [μmol/L]	2nd	15.70	↑	62.70	94.10	0.78	[74]
Vitronectin [ng/mL]	2nd	84.7	↑	70	63.3	0.647	[122]
Afamin [mg/L]	2nd	108.05	↑	44.83	85.00	0.629	[79]
Hematologic markers							
NLR	1st	2.20	↑	66.4	55	0.653	[123]
	2nd	2.34 - 4.27	↑	71.9 – 73.13	68.49 - 84.2	0.696 - 0.867	[124] [125]
SIRI	1st	1.58	↑	67	65	0.71	[126]

Biomarker	Tri-mester	Cut-off	Effect in GDM	Sens.	Spec.	AUC	Reference
	2nd	1.40	↑	85	70	0.833	[75]
SII	1st	875	↑	66	65	0.70	[126]
	2nd	808.83	↑	78	72	0.767	[75]
MPV [fL]	1st	7.38 – 10.1	↑	50 - 69.5	65 – 66.3	0.577 - 0.704	[127] [128]
	2nd	8.0 - 11.05	↑	71.9 - 82	75 - 82.5	0.805 - 0.906	[124] [129]
Plateletcrit [%]	1st	0.22	↑	68.8	51.5	0.628	[127]
	2nd	0.19 - 0.20	↑	62.73 - 77.9	78.18 - 95	0.766 - 0.932	[130] [131]
Neutrophil (10^9/L)	1st	6.46	↑	67	55	0.63	[132]
	2nd	6.46 - 7.85	↑	56.72 - 67	55 - 79.90	0.63 - 0.655	[125] [132]
Platelet (10^9/L)	1st	235	↑	50	71	0.64	[132]
Hb (g/dL)	1st	10.8	↑	87.1	41.6	0.61	[133]
Lymphocyte (10^9/L)	1st	2.09	↑	36	78	0.60	[132]
RBC (10^{12}/L)	1st	4.12	↑	27	87	0.58	[132]
MLR	2nd	0.309	↑	52.09	80.81	0.72	[125]
PLR	2nd	117.46	↑	52.24	69.73	0.623	[125]
WBC (10^9/L)	2nd	10.13	↑	56.72	68.49	0.624	[125]
Platelet distribution width (PDW)	2nd	15.55	↑	-	-	0.584	[99]
Thyroid function markers							
TSH [mIU/L]	1st	5.33	↑	62.9	78.7	0.705	[134]
	2nd	2.58	↑	43.6	92.4	0.71	[135]
TPOAb [IU/mL]	1st	25.01	↑	48.8	85.8	0.642	[136]
	2nd	18.51	↑	39.5	86.7	0.665	[136]
TgAb [IU/mL]	1st	29.60	↑	70.0	80.2	0.793	[136]
	2nd	23.90	↑	51.3	86.2	0.833	[136]
FT3 [pmol/L]	1st	4.61	↑	42.9	80.7	0.724	[136]
FT4 [pmol/L]	2nd	14.00	↓	52.5	71.4	0.626	[136]
Miscellaneous markers							
Serum ferritin [ng/mL]	1st	55.7	↑	62.6	53.7	-	[137]
	2nd	37.55	↑	85.9	81.9	0.904	[138]
Lactoferrin [ng/mL]	1st	794.2	↓	100	95.83	0.98	[139]
SERPINB1 [ng/mL]	2nd	8.75	↑	75.86	81.67	0.832	[79]
Cystatin-C (Cys-C) [mg/L]	2nd	0.95	↑	58.6	73.4	0.722	[140]

Table S2: Biochemical markers for the prognosis of maternal outcomes in women with GDM

Maternal outcomes	Biomarker	Trimester	Cut-off	Effect in GDM	Findings	Reference
Pregnancy-induced hypertension (PIH)	FABP4 [ng/ml]	2nd	-	↑	Increased risk	[141]
	HbA1c (%)	2nd	5.1 - 5.9	↑	Increased risk	[142] [143]
		3rd	5.9	↑	Increased risk	[142]
Preeclampsia	25OHD (ng/mL)	2nd	10	↓	Increased risk	[144]
	Serum ferritin (SF) [ng/mL]	2nd	24.45	↑	Increased risk	[145]
Gestational hypertension	TyG index	1st	-	↑	Increased risk	[89]
Preterm delivery	HbA1c (%)	1st	5.5–6.4	↑	Increased risk	[146]
		2nd	5.5	↑	Increased risk	[143] [147]
	25OHD [ng/mL]	2nd	10	↓	Increased risk	[144]
Cesarean section	HbA1c (%)	2nd & 3rd	5.9	↑	Increased risk	[142] [143]
	TG (mmol/L)	2nd & 3rd	-	↑	Increased risk	[148]
	Glycated albumin (%)	3rd	15.69	↑	Increased risk	[149] [150]
Abnormal amniotic fluid volume	HbA1c (%)	2nd	-	↑	Increased risk	[96]
Postpartum hemorrhage	GlyA (%)	3rd	-	↑	Increased risk	[151]
Shoulder dystocia	HbA1c (%)	2nd	-	↑	Increased risk	[96]
Microalbuminuria	GDF15	2nd	-	↑	Increased risk	[152]

Table S3: Biochemical markers for the prognosis of fetal-neonatal outcomes in women with GDM

Fetal and neonatal outcomes	Biomarker	Trimester	Cut-off	Effect in GDM	Comments	Reference
LGA	HbA1c [%]	1st	5.7	↑	Increased risk	[153]
		2nd	5.7 - 5.9	↑	Increased risk	[142] [153]
		3rd	5.7 - 5.9	↑	Increased risk	[142] [154] [155]
	TG/HDL-C	1st	-	↑	Increased risk	[156]
		2nd	1.85	↑	Increased risk	[110]
	TG [mmol/L]	2nd	3.6	↑	Increased risk	[148] [157]
		3rd	-	↑	Increased risk	[148]
	FPG [mg/dL]	1st	82 – 92.3	↑	Increased risk	[158] [159]
	TyG index	1st	-	↑	Increased risk	[156]
	25(OH)D [ng/mL]	2nd	10	↓	Increased risk	[160]
	pGCD59 [SPU]	1st & 2nd	-	↑	Increased risk	[101]
	GA [%]	3rd	14.1 - 15.8	↑	Increased risk	[154] [155] [161] [162]
	GA/HbA1c	3rd	2.55	↑	Increased risk	[155]
	Fructosamine [μmol/L]	3rd	-	↑	Increased risk	[154]
Macrosomia	Monocyte count	1st	-	↓	Increased risk	[163]
	HbA1c [%]	2nd	5.9	↑	Increased risk	[96] [142] [143]
		3rd	5.9 - 6.1	↑	Increased risk	[142] [164]
	Glycated albumin (GA) [%]	2nd	14	↑	Increased risk	[166]
		3rd	15.69	↑	Increased risk	[149]
	TG [mmol/L]	2nd	-	↑	Increased risk	[148] [165]
		3rd	-	↑	Increased risk	[148]
	Fructosamine [μmol/L]	2nd	200	↑	Increased risk	[167]
	FPG [mg/dL]	3rd	98.6	↑	Increased risk	[149]
Neonatal hypoglycemia	HbA1c [%]	2nd	-	↑	Increased risk	[96]
	Serum ferritin (SF) [ng/mL]	2nd	27.43	↑	Increased risk	[145]
	Glycated albumin [%]	3rd	13.5 - 15.8	↑	Increased risk	[155] [161] [162]
	25-OH-D [ng/mL]	3rd	20	↓	Increased risk	[169]
NICU admission	TG [mmol/L]	2nd & 3rd	-	↑	Increased risk	[148]
	Zonulin [ng/mL]	3rd	-	↑	Increased risk	[170]
Need for neonatal resuscitation	HbA1c [%]	2nd	-	↑	Increased risk	[96]
SGA	Serum ferritin [μg/L]	2nd & 3rd	-	↑	Increased risk	[168]
	25-OH-D [ng/mL]	3rd	20	↓	Increased risk	[169]
Neonatal hyper-bilirubinemia	HbA1c [%]	2nd	-	↑	Increased risk	[147]
	Glycated albumin [%]	3rd	13.7	↑	Increased risk	[155]

Fetal and neonatal outcomes	Biomarker	Trimester	Cut-off	Effect in GDM	Comments	Reference
Neonatal asphyxia	HbA1c [%]	2nd	-	↑	Increased risk	[147]
Neonatal hypocalcemia	Glycated albumin (GA) [%]	3rd	13.9 - 15.8	↑	Increased risk	[155] [161] [162]
Neonatal polycythemia	Glycated albumin (GA) [%]	3rd	14.5 - 14.6	↑	Increased risk	[155] [162]
1st min APGAR score <7	Zonulin [ng/mL]	3rd	-	↑	Increased risk	[117]
Lower 1st min APGAR score	25OHD [ng/mL]	2nd	10	↓	Increased risk	[144]
	MPV [fL]	2nd	-	↑	Increased risk	[129]
	Zonulin [ng/mL]	3rd	-	↑	Increased risk	[170]
Lower 5th min APGAR score	25OHD (ng/mL)	2nd	10	↓	Increased risk	[144]
	Zonulin [ng/mL]	3rd	-	↑	Increased risk	[170]
Respiratory disorders	Serum ferritin (SF) [ng/mL]	2nd	27.37	↑	Increased risk	[145]
	Glycated albumin (GA) [%]	3rd	13.7 - 15.8	↑	Increased risk	[155] [161] [162]
Meconium-stained fluid	Zonulin [ng/mL]	3rd	-	↑	Increased risk	[117] [170]
Myocardial hypertrophy	Glycated albumin [%]	3rd	13.9 - 15.8	↑	Increased risk	[155] [161] [162]
	GA/HbA1c	3rd	2.55	↑	Increased risk	[155]

Table S4: Biochemical markers for the prognosis of long-term outcomes in women with GDM

Postpartum outcomes	Biomarker	Trimester	Cut-off	Effect in GDM	Comments	Reference
Postpartum abnormal glucose metabolism	TG [mmol/L]	2nd	2.89	↑	Increased risk	[171]
Postpartum glucose intolerance	TPOAb [IU/mL]	1st	-	↑	Increased risk	[136]
	TgAb [IU/mL]	1st	-	↑	Increased risk	[136]
	Hb A1c [%]	2nd	5.55	↑	Increased risk	[96]
	Prolactin (PRL) [ng/mL]	1st & 2nd	115	↓	Increased risk	[173]
	Vitamin D [ng/mL]	2nd & 3rd	20	↓	Increased risk	[174]
Postpartum prediabetes	PEDF [µg/mL]	2nd	-	↑	Increased risk	[175]
Postpartum diabetes mellites (PDM)	Betatrophin [pg/mL]	2nd	-	↑	Increased risk	[68]
	Hb A1c [%]	2nd	5.5	↑	Increased risk	[172]
	PEDF [µg/mL]	2nd & 3rd	4.23	↑	Increased risk	[176]
